# Role of Healthy Lifestyle and Diet Quality in the Development of Colorectal Cancer in the Adult Population in the Kurdistan Region: A Case-Control Study

**DOI:** 10.7759/cureus.58764

**Published:** 2024-04-22

**Authors:** Ayid M Qasim, Sardar H Arif

**Affiliations:** 1 Infection Control, Duhok General Directorate of Health, Duhok, IRQ; 2 Surgery, University of Duhok, Duhok, IRQ

**Keywords:** diet quality, healthy eating index, healthy lifestyles, risk factors, colorectal cancer

## Abstract

Background

The incidence of colorectal cancer (CRC) is increasing in developing countries. The factors contributing to the risk of CRC are not known in developing countries. Therefore, this study aimed to explore the role of a healthy lifestyle on CRC in the adult population in the Kurdistan Region of Iraq.

Methodology

In this case-control investigation, patients previously diagnosed with CRC were included as cases (n = 84) and the healthy adult population as healthy controls (n = 87). The patients were selected from the Gastroenterology Unit of Azadi Teaching Hospital and Emergency Teaching Hospital. The healthy controls were selected from the caregivers of patients who met the eligibility criteria.

Results

Individuals with a history of chronic disease (63.08% vs. 40.52%; p = 0.0043), a history of hypertension (71.74% vs. 40.80%; p = 0.0003), and a history of inflammatory bowel disease (IBD) (59.42% vs. 42.16%; p = 0.0267) had a significantly higher prevalence of CRC compared to healthy controls. CRC patients had significantly lower diet quality scores than healthy controls (36.27 vs. 37.83; p = 0.0002). The study showed that CRC patients had a significantly lower lifestyle index score compared to healthy controls (10.20 vs. 11.69; p = 0.0002). In addition, CRC patients had lower scores for diet (0.42 vs. 1.00; p < 0.0001), smoking (2.92 vs. 4.0; p < 0.0001), and physical activity (1.02 vs. 1.70; p < 0.0001) compared to healthy controls. However, CRC patients and healthy controls had similar alcohol index scores (5.0 vs. 530; p = 1.000) and body mass index (1.04 vs. 1.01; p = 0.8982).

Conclusions

This study showed that CRC was associated with having a history of bad diet quality and unhealthy lifestyles. In addition, a history of chronic diseases, hypertension, and IBD was associated with the risk of CRC.

## Introduction

Colorectal cancer (CRC) is the third most prevalent cancer among men, comprising roughly 10.0% of all cancer cases, with an estimated annual incidence of 746,000 cases. Similarly, among women, CRC is the second most common cancer type, accounting for around 9.2% of total cancer cases, with approximately 614,000 new cases reported annually. Geographic variations exist in CRC incidence, with developed regions witnessing approximately 55% of global cases [1]. The correlation between increased CRC prevalence and westernization underscores the significance of lifestyle in disease etiology [2]. This hypothesis is further supported by the World Cancer Research Fund/American Institute for Cancer Research expert panel, which acknowledges that maintaining an active lifestyle and consuming diets rich in fiber, fish, dairy products, nuts and fruits, and vegetables are associated with a reduced risk of CRC. Conversely, factors such as higher body mass index (BMI), smoking, increased waist circumference, alcohol intake, and the consumption of red and processed meats are linked to an elevated risk of CRC [3].

As previously indicated, CRC incidence exhibits notable variations across different countries [4]. In recent years, a concerning trend has emerged in developing countries like Iran, where the incidence of CRC has escalated. In Iran, CRC has risen to become the third most prevalent cancer among men and the fourth most prevalent among women [5]. In the Kurdistan Region, CRC ranks prominently among cancer types. CRC was identified as the third most common cancer in women and the fifth most common in men in the Erbil Governorate between 2013 and 2019. Furthermore, CRC is the fifth most common cancer in males and females in the Duhok Governorate [6].

The development of CRC is influenced by various factors, categorized into two types. Non-modifiable factors, such as a family history of the disease, inflammatory bowel disease (IBD), genetics, advancing age, and being male, play significant roles in predisposing individuals to CRC. Conversely, modifiable risk factors, such as smoking, dietary habits, physical inactivity, and obesity, offer avenues for intervention and prevention. Adenomatous polyps (adenomas), characterized by abnormal proliferation of epithelial cells in the colon and rectum, are implicated in approximately 90% of CRC cases, with a potential for malignant transformation [4]. The rising incidence of CRC in the Kurdistan Region of Iraq may be attributed to shifts in lifestyle and dietary factors, resembling Westernized lifestyles. Factors such as smoking and obesity, identified as components of this Westernized lifestyle, could contribute to the increasing prevalence of CRC [7].

Numerous studies have investigated individual health behaviors while treating other lifestyle factors as mere covariates. However, it is imperative to recognize that individuals do not exist in isolation, and their behaviors are interconnected within a multidimensional lifestyle framework. Approaching research in this manner provides a more comprehensive understanding of the disease etiology and offers a more logical path for disease prevention. Additionally, utilizing techniques such as estimating health impact measures such as population-attributable risks enhances decision-making in public health [8]. Furthermore, epidemiological data are scarce on CRC risk factors, particularly in the Kurdistan Region. As highlighted by M-Amen et al. in 2022, and Ali et al. in 2022, this gap underscores the need for further research and surveillance efforts to elucidate the specific risk factors contributing to CRC incidence in the region [6,9].

This study aimed to examine the effect of a healthy lifestyle index and diet quality on CRC in adult patients in the Kurdistan Region of Iraq. We hypothesized that CRC patients were more likely to have a history of low diet quality, lower levels of physical activity, and high levels of smoking compared to healthy controls.

## Materials and methods

Study design

In this case-control study, patients previously diagnosed with CRC were considered cases, and the healthy adult population was considered healthy controls. Patients who attended the Gastroenterology Unit of Azadi Teaching Hospital and Emergency Teaching Hospital were screened by a surgeon (second author) and the first researcher for the eligibility criteria. Healthy controls were selected from the caregivers of the patients who met the eligibility criteria. The study outcomes were measured in both CRC patients and healthy controls.

Study setting

CRC patients in this study were selected from patients diagnosed with CRC at Azadi and Emergency Teaching hospitals in Duhok City. These two hospitals were selected because the majority of patients visit these two hospitals for CRC. To meet the required number of patients, patients were included in some private clinics as well. As the sociodemographic characteristics of the patients who visited the public hospitals and private clinics could be different, we included the patients from both the public and private sectors to include a representative sample of the target population. Healthy individuals were selected from the caregivers of the patients as the caregivers and patients would have approximately the same sociodemographic characteristics. Therefore, the case and control groups were homogenous in terms of age, gender, education, and other related characteristics. The patients were included from the first of July 2022 to the end of December 2023.

Sampling technique

The required sample for this study was recruited through the purposive sample technique. Patients previously diagnosed with CRC were selected purposively from the general public hospitals and private clinics. Patients who met the inclusion and exclusion criteria were included in this study. The medical records of the patients diagnosed with CRC were drawn from the corresponding settings in Duhok City to include the general and medical information of the patients.

Inclusion and exclusion criteria

Patients diagnosed with CRC of both genders irrespective of sociodemographic aspects were eligible for this study. Patients diagnosed with other cancers along with CRC were excluded from the study. Patients with >20% missing information were excluded as well (two cases in this study). Healthy individuals were the caregivers of cases to ensure homogeneity for sociodemographic aspects.

Instruments for data collection

Data were collected through the use of a questionnaire developed through a broad review of previous studies in the literature. The questionnaire included two parts for data collection. The first one dealt with related patients’ general characteristics such as age, sex, marital status, socioeconomic status, level of education, occupation, income, family history of CRC, history of chronic diseases, history of current chronic diseases, and residence. The second part dealt with patients’ healthy lifestyle factors. Healthy lifestyle factors consisted of five healthy lifestyles, namely, smoking status, alcohol intake, physical activity, and diet quality.

Healthy lifestyle

The following variables were included for measuring the Healthy Lifestyle Index Score (HLIS): diet, smoking, alcohol consumption, BMI, and physical activity. This scale was used to define “healthier” and “less healthy” behaviors for each lifestyle risk factor. The HLIS scale consisted of five lifestyle risk factors. Each component was rated as 0 for the healthiest lifestyle and 4 for the unhealthiest lifestyle. The total score ranged between 0 and 40. The higher the score the healthier the lifestyle and better adherence to healthy lifestyle behaviors. The diet in this scale was made using the items of the Diet Habits Questionnaire (DHQ) [10]. In the HLIS, the highest 20% of the responses were devoted to higher scores of 4, and the lowest 20% of the responses corresponded to the lowest score of zero. In this scale, the scores were defined as 0 (<68 in total DHQ score), 1 (68-76), 2 (77-83), 3 (84-90), and 4 (91-100) [11].

For BMI, the scores were defined as 0 (BMI >29 kg/m^2^), 1 (26-28 kg/m^2^), 2 (24-25 kg/m^2^), 3 (22-23 kg/m^2^), and 4 (<22 kg/m^2^). Regarding physical activity, the scale used the metabolic equivalent task (MET) minutes/week and was converted to MET hours/week. In the scale, the physical activity scores were 0 (<45 MET/week), 1 (45-68 MET/week), 2 (69-95 MET/week), 3 (96-133 MET/week), and 4 (134 MET/week and higher). Regarding smoking, the healthiest smoking behavior was determined as “never smoked” with a score of 4. The current (current smokers who smoked >15 cigarettes/day) received a score of 0. Current smokers who smoked ≤15 cigarettes/day received a score of 1. Ex-smokers who quit less than 10 years ago received a score of 2. Ex-smokers who quit 10 years or more ago received a score of 3. Regarding alcohol consumption, the score of 0 was defined as consumption of an average ≥20 g/day of ethanol. Individuals who consumed ≥10 and <20 g/day received a score of 1. Individuals who consumed an average of ≥5 and <10 g/day received a score of 2. Individuals who consumed an average of >0 and <5 g/day received a score of 3. Non-alcoholic individuals received a score of 4 [11].

Diet quality

The Diet Quality Index (DQI) scale assessed the diet quality of patients and healthy individuals by considering various dietary indicators. These indicators encompass a range of food groups, including bread, vegetables/salads, fruits, yogurt or milk, pasta or rice, olive or sunflower oil, alcoholic beverages, breakfast flakes, red meat, sausages, cheese, pastry or sweets, butter or lard, other vegetable oils (one tablespoon), fast food, fish, legumes, and nuts. Each indicator is rated on a scale from 1 to 3, reflecting the frequency of consumption. In the second category, the consumption of foods considered detrimental was scored 2 if reported four to six times per week, with more and less frequent consumption earning scores of 1 and 3, respectively. Conversely, in the third category, high consumption (four or more times per week) of foods considered beneficial was awarded a score of 3, while intakes of two to three times and less than twice a week received scores of 2 and 1, respectively. The total DQI score was calculated by summing up the scores for all food items, resulting in a possible score ranging from 18 to 54. This comprehensive scoring system allowed for a nuanced evaluation of dietary habits and their impact on overall diet quality [12].

Methods of data collection

The data were collected through direct face-to-face interviews with the patients or their caregivers as appropriate. The researcher administered the questionnaire through a self-reported technique.

Data analysis

The general and medical characteristics of CRC patients and healthy controls are presented as mean and standard deviation or number and percentage. The independent t-test and chi-square tests were performed to assess homogeneity between CRC patients and healthy controls. The comparisons of medical characteristics between healthy controls and CRC patients were examined using chi-square tests. Comparisons of diet quality scores between healthy controls and CRC patients were examined using an independent t-test. Comparisons of diet quality and healthy lifestyles index scores of patients with different enteral and medical characteristics were examined using an independent t-test and one-way analysis of variance tests. A p-value <0.05 was considered significant. The statistical calculations were performed using JMP® version 17.1.0 (SAS Institute Inc., Cary, NC, USA) (https://www.jmp.com/en_us/home.html).

Ethical considerations

The ethical approval for this study was obtained from the local health ethics committee, the Scientific Research Division, on 13, July 2021 (reference number: 13072021-7-21). In addition, we obtained permission from the Azadi Teaching Hospital and Duhok Emergency Hospital. In this study, patients and healthy controls who did not permit us to include the study were excluded.

## Results

The mean age of the CRC patients (n = 84) and healthy controls (n = 87) was 53.37 and 50.39 years, respectively, with no statistically significant difference (p = 0.1251). CRC patients and healthy controls were similar in sex (p = 0.4161), marital status (p = 0.8237), residency (p = 0.9091), education (p = 0.6278), and occupation (p = 0.5306). However, CRC patients and healthy controls had significant differences in income level (Table 1).

**Table 1:** General characteristics of healthy controls and colorectal cancer patients. ^a^: independent t-test; ^b^: chi-square tests were performed for statistical analyses. SD = standard deviation; SEM = standard error of the mean

General characteristics	Study groups, n (%)		P-value (two-sided)
	Healthy controls (n = 87)	Colorectal cancer patients (n = 84)	
Age, mean (SD)	50.39 (11.25)	53.37 (13.92)	0.1251^a^
Range	27-76	18-81	
SEM	1.21	1.52	
Age groups (years)			
20–29	2 (2.30)	5 (5.95)	0.1135^b^
30–39	11 (12.64)	11 (13.10)	
40–49	25 (28.74)	11 (13.10)	
50–59	30 (34.48)	28 (33.33)	
60–69	15 (17.24)	22 (26.19)	
70 and older	4 (4.60)	7 (8.33)	
Sex			
Male	54 (62.07)	47 (55.95)	0.4161^b^
Female	33 (37.93)	37 (44.05)	
Marital status			
Single	7 (8.05)	6 (7.14)	0.8237^b^
Married	80 (91.95)	78 (92.86)	
Residency			
Rural	37 (42.53)	35 (41.67)	0.9091^b^
Urban	50 (57.47)	49 (58.33)	
Income			
Low	41 (47.13)	23 (27.38)	0.0218^b^
Intermediate	45 (51.72)	58 (69.05)	
High	1 (1.15)	3 (3.57)	
Education			
Illiterate/Read and write	37 (42.53)	43 (51.19)	0.6278^b^
Under high school	35 (40.23)	27 (32.14)	
High school	4 (4.60)	5 (5.95)	
College	11 (12.64)	9 (10.71)	
Occupation			
Housewife/retired/unemployed	86 (98.85)	80 (95.24)	0.5306^b^
Administrative job	0 (0.00)	1 (1.19)	
Agricultural job	0 (0.00)	1 (1.19)	
Business job	0 (0.00)	1 (1.19)	
Healthcare worker	1 (1.15)	1 (1.19)	

The risk of CRC patients was not associated with a family history of CRC (60.0% vs. 48.45%; p = 0.5305), a history of diabetes mellitus (58.62% vs. 47.18%; p = 0.2616), a history of ischemic heart disease (IHD) (62.50% vs. 48.47%; p = 0.4910), history of other chronic diseases (50.0% vs. 49.11%; p = 1.000), history of polyps (100% vs. 48.52%; p = 0.2398), history of gastritis (61.90% vs. 47.33%; p = 0.2109), history of colonoscopy (62.50% vs. 48.47%; p = 0.4910), and history of sigmoidscopy (50.0% vs. 49.11%; p = 1.000). However, individuals with a history of chronic disease (63.08% vs. 40.52%; p = 0.0043), a history of hypertension (71.74% vs. 40.80%; p = 0.0003), and a history of IBD (59.42% vs. 42.16%; p = 0.0267) had a significantly higher prevalence of CRC compared to healthy controls (Table 2).

**Table 2:** Comparison of medical characteristics between healthy controls and colorectal cancer patients. Chi-square tests were performed for statistical analyses. OR = odds ratio; CI = confidence interval; CRC = colorectal cancer; DM = diabetes mellitus; HTN = hypertension; IHD = ischemic heart disease; VHD = valvular heart disease; IBD = inflammatory bowel disease

Medical characteristics	Study groups, n (%)		OR (95% CI)	P-value (two-sided)
	Healthy controls (n = 87)	Colorectal cancer patients (n = 84)		
Family history of CRC				
No	83 (51.55)	78 (48.45)	Reference	0.5305
Yes	4 (40.00)	6 (60.00)	1.6 (0.43-5.87)	
History of chronic disease				
No	63 (59.43)	43 (40.52)	Reference	0.0043
Yes	24 (36.92)	41 (63.08)	2.5 (1.33-4.73)	
History of DM				
No	75 (52.82)	67 (47.18)	Reference	0.2616
Yes	12 (41.38)	17 (58.62)	1.59 (0.71-3.56)	
History of HTN				
No	74 (59.20)	51 (40.80)	Reference	0.0003
Yes	13 (28.26)	33 (71.74)	3.68 (1.77-7.68)	
History of IHD				
No	84 (51.33)	79 (48.47)	Reference	0.4910
Yes	3 (37.50)	5 (62.50)	1.77 (0.41-7.66)	
History of VHD				
No	86 (50.59)	84 (49.41)	Reference	1.000
Yes	1 (100)	0 (0.0)	1.04 (0.06-16.84)	
History of other chronic diseases				
No	86 (50.89)	83 (49.11)	Reference	1.000
Yes	1 (50.0)	1 (50.0)	1.04 (0.06-16.84)	
History of IBD				
No	59 (57.84)	43 (42.16)	Reference	0.0267
Yes	28 (40.58)	41 (59.42)	2.01 (1.08-3.74)	
History of polyps				
No	87 (51.48)	82 (48.52)	Reference	0.2398
Yes	0 (0.0)	2 (100)	2.1 (0.19-23.58)	
History of gastritis				
No	79 (52.67)	71 (47.33)	Reference	0.2109
Yes	8 (38.10)	13 (61.90)	1.81 (0.71-4.62)	
History of colonoscopy				
No	84 (51.53)	79 (48.47)	Reference	0.4910
Yes	3 (37.50)	5 (62.50)	1.77 (0.41-7.66)	
History of sigmoidoscopy				
No	86 (50.89)	83 (49.11)	Reference	1.0000
Yes	1 (50.0)	1 (50.0)	1.04 (0.06-16.84)	

CRC patients had significantly lower diet quality scores compared to healthy controls (36.27 vs. 37.83; p = 0.0002). CRC patients had a significantly lower HLIS compared to healthy controls (10.20 vs. 11.69; p = 0.0002). In addition, CRC patients had lower scores for diet (0.42 vs. 1.00; p < 0.0001), smoking (2.92 vs. 4.0; p < 0.0001), and physical activity (1.02 vs. 1.70; p < 0.0001) compared to healthy controls. However, both CRC patients and healthy controls had similar scores of alcohol index (5.0 vs. 530; p = 1.000) and BMI (1.04 vs. 1.01; p = 0.8982) (Table 3).

**Table 3:** Comparison of the diet quality score between healthy controls and colorectal cancer patients. An independent t-test was performed for statistical analyses CI = confidence interval; SD = standard deviation; SEM = standard error of the mean; BMI = body mass index; HLIS = Healthy Lifestyle Index Score

Outcomes	Study groups			P-value (two-sided)
	Healthy controls (n = 87)	Colorectal cancer patients (n = 84)	Mean difference (95% CI)	
Diet quality score				
Mean (SD)	37.83 (2.39)	36.27 (2.67)	1.31 (0.62 to 1.99)	0.0002
Range	30–43	29–42		
SEM	0.26	0.29		
HLIS score				
Mean (SD)	11.69 (2.77)	10.20 (2.24)	1.49 (0.72 to 2.26)	0.0002
Range	3-19	2-15		
SEM	0.30	0.25		
HLIS items				
Diet	1.00 (1.03)	0.42 (0.62)	0.58 (0.32 to 0.84)	<0.0001
Smoking	4 (0.0)	2.92 (1.58)	1.08 (0.75 to 1.42)	<0.0001
Alcohol intake	5.0 (0.0)	5.0 (0.0)	NA	1.0000
BMI	1.01 (1.25)	1.04 (1.22)	-0.02 (-0.40 to 0.35)	0.8982
Physical activity	1.70 (1.00)	1.02 (0.93)	0.68 (0.39 to 0.97)	<0.0001

The diet quality of CRC patients with a history of IHD (34.20 vs. 36.67; p = 0.0237) and a history of colonoscopy (34.60 vs. 36.75; p = 0.0373) was significantly lower compared to healthy controls. The diet quality of CRC patients was not significantly different in patients with other medical and general characteristics (Table 4).

**Table 4:** Comparison of diet quality with general and medical characteristics of colorectal cancer patients. SD = standard deviation; SEM = standard error of the mean; CRC = colorectal cancer; DM = diabetes mellitus; HTN = hypertension; IHD = ischemic heart disease; IBD = inflammatory bowel disease

Characteristics	Diet quality			P-value (two-sided)
	Mean	SD	SEM	
Age groups (years)				
20–29	35.4	3.58	1.6	0.7168
30–39	35.64	2.87	0.87	
40–49	36.55	3.30	0.99	
50–59	36.78	1.67	0.32	
60–69	36.14	2.96	0.63	
70 and older	37.00	1.73	0.65	
Sex				
Male	36.60	2.27	0.35	0.9733
Female	36.62	2.24	0.37	
Education				
Illiterate/read and write	36.65	2.76	0.42	0.2579
Under high school	36.46	1.68	0.33	
High school	36.20	3.63	1.62	
College graduates	34.78	2.91	0.97	
History of CRC				
No family history	36.45	2.09	0.24	0.1069
Second degree	38.20	4.92	2.20	
History of chronic disease				
No	37.02	2.21	0.34	0.0921
Yes	36.18	2.22	0.36	
History of DM				
No	36.78	2.13	0.27	0.0536
Yes	35.53	3.04	0.74	
History of HTN				
No	36.90	2.19	0.31	0.1534
Yes	36.16	2.28	0.41	
History of IHD				
No	36.67	2.18	0.25	0.0237
Yes	34.20	4.15	1.85	
History of IBD				
No	36.29	2.25	0.35	0.1926
Yes	36.95	2.21	0.35	
History of gastritis				
No	36.45	2.26	0.27	0.8533
Yes	36.31	3.68	1.02	
History of colonoscopy				
No	36.75	2.11	0.24	0.0373
Yes	34.60	3.36	1.50	

Middle-aged participants had higher HLIS scores compared to the younger and older age groups (p = 0.0026). In addition, female patients had higher HLIS compared to male patients (11.24 vs. 9.22; p < 0.0001). The HLIS was not significantly different in patients with other general and medical characteristics (Table 5).

**Table 5:** Comparison of the Healthy Lifestyle Index Score with general and medical characteristics of colorectal cancer patients. SD = standard deviation; SEM = standard error of the mean; CRC = colorectal cancer; DM = diabetes mellitus; HTN = hypertension; IHD = ischemic heart disease; IBD = inflammatory bowel disease

Characteristics	Healthy Lifestyle Index Score			P-value (two-sided)
	Mean	SD	SEM	
Age groups (years)				
20–29	9.20	1.92	0.86	0.0026
30–39	11.55	1.86	0.56	
40–49	11.40	1.17	0.37	
50–59	10.96	1.27	0.26	
60–69	9.24	2.47	0.54	
70 and older	10.00	1.91	0.72	
Sex				
Male	9.22	2.54	0.37	<0.0001
Female	11.24	1.38	0.23	
Education				
Illiterate/read and write	10.56	1.88	0.29	0.1560
Under high school	9.67	2.83	0.54	
High school	8.4	2.07	0.93	
College graduates	9.44	3.57	1.19	
History of CRC				
No	10.22	2.22	0.26	0.4293
Yes	9.40	2.61	1.17	
History of chronic disease				
No	9.93	2.67	0.41	0.4427
Yes	10.33	1.90	0.30	
History of DM				
No	9.91	2.65	0.32	0.4077
Yes	10.47	1.59	0.38	
History of HTN				
No	10.18	2.40	0.34	0.9395
Yes	10.22	2.00	0.35	
History of IHD				
No	10.23	2.26	0.26	0.5424
Yes	9.60	1.82	0.81	
History of IBD				
No	10.21	2.38	0.36	0.9524
Yes	10.18	2.10	0.34	
History of gastritis				
No	10.19	2.25	0.27	0.9505
Yes	10.23	2.24	0.62	
History of colonoscopy				
No	10.13	2.26	0.26	0.6358
Yes	9.60	4.39	1.96	

## Discussion

This study showed that CRC patients had a worse history of diet quality and healthy lifestyles compared to healthy controls. Moreover, CRC was associated with a history of chronic disease, hypertension, and IBD.

Studies performed in other countries have shown that low diet quality is related to the risk of CRC. For example, a large case-control study [13] included 1,144 patients diagnosed with CRC and 60,549 individuals with no diagnosed disease. The study reported that hyperlipidemia, a history of IBD and polyps, and a history of diabetes were associated with the risk of CRC. The risk of CRC development was shown to be associated with low scores on the Healthy Lifestyles Index and Comorbidity History Index. Other studies have linked lifestyle factors to the risk of CRC [14-17]. A larger European cohort study examined a combination of five healthy lifestyle behaviors. Healthy weight, non-smoking, limited alcohol intake, physical activities, and a healthy diet contributed to a lower risk of CRC [14]. The designed protective lifestyle factor index was shown to relate to reduced age- and sex-standardized incidence of CRC [16].

Alcohol consumption is a significant factor in colorectal oncogenesis [13]. This kind of association has been confirmed in the meta-analysis. Keivanlou et al. [18] included 11 studies in a review and showed a significant positive relationship between alcohol intake and CRC risk (odds ratio (OR) = 1.51; 95% confidence interval (CI) = 1.03-2.22; p = 0.03). A dose-response meta-analysis of 16 published cohort studies included more than 6,300 CRC patients. The review reported that high alcohol consumption is associated with a risk of colon (relative risk (RR) = 5 1.50; 95% CI = 1.25, 1.79) and rectal cancer (RR = 1.63; 95% CI = 1.35, 1.97) compared to lower intake of alcohol. The risk of colon or rectal cancer increased by 15% for each 100 g of alcohol intake/week [19]. This association was not found in our study because the incidence of alcohol intake is low in our region due to religious prohibitions. The exact mechanism of the role of alcohol consumption on CRC risk is not fully known. However, the plausible events include a genotoxic effect of acetaldehyde, the main metabolite of ethanol [20]. CRC has been shown to increase heterozygous carriers of a specific allele of the *ALDH2* gene [21]. ALDH2*2 is common in Asian populations, but roughly all Europeans are homozygous for the ALDH2*1 allele [20]. A study recommended that polymorphism in the *ADH3* gene may modify the alcohol-CRC risk association [22].

Our study showed that a history of IBD is associated with a risk of CRC. Other studies have reported a significant association between a history of IBD and CRC incidence. Some of these studies have included some other comorbidities such as hypertension, diabetes, and hyperlipidemia. The history of IBD, colorectal polyps, and diabetes contribute to an increased risk of CRC development. The imbalance between pro- and anti-inflammatory cytokines (interleukin-6, tumor necrosis factor-alpha, etc.), oxidative DNA damage, and genomic instability have implications for colorectal carcinogenesis in IBD patients [23]. Epidemiological studies have reported that the risk of CRC is associated with a history of gastritis, schistosomiasis, hypertension, hyperlipidemia, and CRC risk [24-26].

The regression analysis showed that the higher Healthy Eating Index score (OR = 0.04; 95% CI = 0.01-0.12) and the Mediterranean-Style Dietary Pattern score (OR = 0.19; 95% CI = 0.09-0.38) were associated with lower odds of CRC. Further, the higher Healthy Eating Index score (OR = 0.04; 95% CI = 0.08-0.32) and Mediterranean-Style Dietary Pattern Score (OR = 0.04; 95% CI = 0.08-0.32) were associated with a lower risk of colorectal adenomas [4]. These findings are consistent with the results of our study and other published studies in the literature [27-29]. Finally, postoperative sepsis after colorectal surgery should not be overlooked in CRC patients [30], specifically among patients aged 70 years and older [31].

Strengths and limitations of the study

A strength of this study is that we tried to include the available and accessible patients in the study, as much as possible. In addition, we ensured homogeneity for several factors (age, gender, education, occupation, etc.) between CRC patients and controls. However, the study was not exempt from limitations. First, healthy lifestyle factors were documented through a self-reported technique with a possibility for recall bias in this case-control study. Second, the patients included in this study may not be representative of the patients in this region.

## Conclusions

This study showed that CRC was associated with a worse history in terms of diet quality and unhealthy lifestyles. A history of chronic diseases, hypertension, and IBD were associated with the risk of CRC in this study. However, a family history of CRC, diabetes mellitus, IHD, valvular heart disease, other chronic diseases, polyps, gastritis, and colonoscopy was not associated with CRC.
